# Shielding effects in polymer–polymer reactions. V. Concentration dependence of contact formation between star-branched and linear chains^☆^

**DOI:** 10.1016/j.polymer.2013.05.055

**Published:** 2013-07-19

**Authors:** Michael M. Nardai, Gerhard Zifferer

**Affiliations:** Department of Physical Chemistry, University of Vienna, Währinger Str. 42, A-1090 Wien, Austria

**Keywords:** Polymer reaction, Dissipative Particle Dynamics, Shielding factor

## Abstract

By use of the Dissipative Particle Dynamics (DPD) simulation technique mixtures of star-branched (arm number *F* = 4) and linear chains in athermal (good) solvent are analyzed regarding probabilities for intermolecular contacts of various reactive sites within different polymer coils. The accompanying sterical hindrances are described in the framework of shielding factors in order to investigate reactions and side reactions in radical polymerization and other techniques that involve polymer–polymer coupling. The shielding factors are studied as a function of total concentration from high dilution up to the bulk for different chain lengths of star-shaped and linear chains. Results indicate that their concentration dependence can be described by a power law for systems above the overlap concentration, whereas the chain length dependence vanishes when extrapolating to infinite chain lengths in that concentration range. Also the influence of the ratio of star chains and linear chains is studied for various concentrations.

## Graphical abstract

**Figure:**
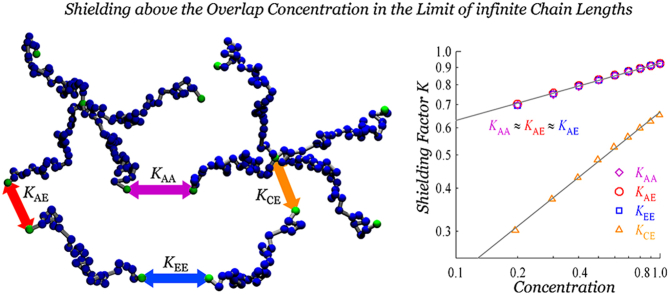

## 1 Introduction

In many polymerization techniques, the reacting groups are each connected to different polymer coils. For example, the key step during reversible addition-fragmentation chain transfer (RAFT) polymerization [1–6] is that chains undergoing radical polymerization are attached to and detached from stabilizing groups in an equilibrium reaction. For Z-RAFT [7] polymerization this stabilizing group comprises the center of a star-shaped polymer and growing linear chains carrying a radical end have to diffuse through the concentration field of star arms attached to the reactive center. In this process the surrounding free chains are partly hindered from approaching and this effect gets more pronounced with increasing chain lengths during polymerization. These effects can be quantified by introducing the so called shielding factors *K*_*ij*_, being proportional to the rate constant *k*_*ij*_ of a polymer–polymer reaction with two reactive sites *i* and *j* located along each chain, normalized by the limiting case of *k*_*ij*_ with chain length one – i.e., *k*_0_ of the reactive sites not being attached to chains [8]. Therefore, a low value of *K*_*ij*_ indicates pronounced shielding and vice versa. Depending on the choice of *i* and *j*, values of *K*_*ij*_ = *k*_*ij*_/*k*_0_ describe shielding of the desired step, being it center-end contacts, for the Z-RAFT scheme, or side reactions like transfer to polymer [9], star–star coupling for R-RAFT [10–13], radical addition to RAFT-groups [14,15] and other undesired recombinations of radical chains [16–19] which lead to dropping rate constants or higher polydispersities.

In the previous contributions various shielding effects have been investigated for contact formations between several types of equivalent chains. Addressing the limit of infinite dilution, single pairs of coils have been studied thoroughly by means of the Monte Carlo and Exact Enumeration (MC + EE) technique: shielding factors between chain ends and star centers of 4-arm stars including variation of chain stiffness [20], then expanding the studies to 6-arm stars and different locations of reactive sites along the chains [21], and describing various contact formations between stars of different arm number and chain length [22]. Eventually, effects of different solvent qualities [23] have been investigated by MC + EE, as well as by Dissipative Particle Dynamics (DPD) [24,25]: As a mesoscopic molecular dynamics approach with hydrodynamic interactions included, it is also used in this study to simulate the interpenetration of polymer coils directly as a function of time. Both simulation methods yielded similar results, despite their conceptually different approach. This indicates that the effect of (partly hindered) segmental diffusion of the reacting groups leading to the shielding effect is reflected in both techniques and segmental diffusion is slow enough so that contacts are formed from equilibrium pair configurations. By changing the interaction parameters from athermal (no enthalpy change of solution, i.e., good solvent) to bad solvent qualities, an increase of the shielding factors (i.e., decreasing shielding) has been observed, with a maximum in the vicinity of the parameter for the theta condition. In this solvent regime, self avoiding chains scale as ideal chains, because their total excluded volume is zero by compensation of positive and negative contributions. Although these coils are reduced in size (measured by their mean square dimensions) and, therefore, show a higher segmental density they are also effectively drawn together by the surrounding bad solvent, which facilitates the contact formation of reactive sites. Following these findings, experiments have been performed, proving the suggestions deducted from simulation data. Indeed, the polydispersity of the generated polymer could be reduced by applying theta solvent conditions (instead of good solvents) during the reaction process [23].

From theory it is well known that properties of polymers also change toward theta behavior when the concentration of coils is increased [26]. Eventually, in the bulk, global properties and pair distribution functions are similar as in theta solutions. Therefore, it is straightforward to investigate not only the influence of solvent quality on shielding but also the concentration of reacting species, which is the main topic of this contribution.

When increasing the concentration of stars and chains not only scaling and shrinkage have to be taken into account, but also further effects caused by concentration dependent effective interactions [27]: Mixtures of linear chains and stars have been studied theoretically, experimentally and by simulations, describing regions of cluster formation and polymer glasses in the corresponding phase diagrams [28,29]. In this study we avoid these additional effects (which would greatly increase the parameter space to be covered in order to give consistent results) restricting the functionality of stars to a relatively small value compared to those studies. Thus, our systems are well below the critical functionality described in Ref. [30] that would lead to cluster formation, phase separation, etc. for mixtures as well as for pure star polymer solutions.

For higher concentrations, DPD is a suitable technique since simulations ranging from dilute over semi-dilute up to the solvent free bulk are possible and easily implemented. (The latter concentration regime poses problems within MC simulation because most efficient algorithms rely on free space between chains in order to perform relaxation trials, although bond breaking algorithms [31] or cooperative motion schemes [32] are used in this latter case.)

**Scheme 1:**
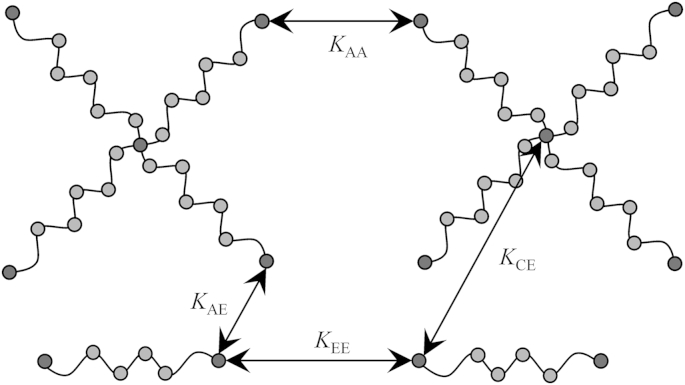
Graphic representation of two star chains and two linear chains; Arrows connecting reactive sites show the combinations evaluated concerning pair distribution and shielding factors in this contribution.

In the present contribution we simulate mixtures of DPD chains with star topologies (arm number *F* = 4) and linear chains with the same chain length as one arm of a star. For each set of chain lengths the total concentration *Φ* is varied from low values beneath the overlap concentration *Φ*∗ up to the solvent free bulk. Of course, for every polymerization technique there is a concentration range that yields optimal results, so data for low concentrations are applicable for, e.g., RAFT polymerization, for which high dilutions are necessary to counter recombination and transfer reactions, whereas data for high concentrations should contribute to cases of coupling reactions up to the melt state in bulk polymerization. Therefore, for the whole concentration range shielding factors between ends of linear chains, ends of star arms, ends of arms and linear chains, as well as between centers of stars and ends of linear chains are investigated, see Scheme 1.

## 2 Computational method

Dissipative Particle Dynamics (DPD) [24,25] is a coarse grained molecular dynamics technique used to calculate properties of fluids in the mesoscale region which, contrary to Monte Carlo approaches, explicitly calculates the motion of repulsive solvent point masses and their effect on polymers immersed in these “solvent beads”. The polymers are treated as equivalent chains consisting of similar point masses, which are connected by spring forces. To avoid artificial surface effects at the boundaries of a simulated volume, periodic boundary conditions in all directions are used. The proper choice of interaction parameters determines the repulsive forces between beads which allow for adjusting the solvent quality.

The system is propagated in time by solving Newton's equations of motion. The algorithm closely follows the original one, see Ref. [33]. Calculations are performed in a dimensionless representation. Therefore, all distances given in the following graphs are in multiples of the cutoff radius *r*_C_ of the repulsive potential. Unlike atomistic force fields, the interaction potential contains stochastic and friction terms as a result of a formal coarse graining procedure [34], yielding a canonical ensemble. Still all force contributions act pair wise (action = reaction) and together with the explicit treatment of solvent, momentum transport within the fluid is possible. This ensures correct simulation of hydrodynamic interaction [35]. The DPD algorithm reads as follows: Propagation of particle *i* under the influence of a pair wise interaction with particle *j*,

(1) $$\frac{\mathrm{d}r_{i}}{\mathrm{d}t}=v_{i}\text{,}\;\frac{\mathrm{d}v_{i}}{\mathrm{d}t}=f_{i}=\sum_{j\neq i}\left(f_{ij}^{\text{c}}+f_{ij}^{\text{d}}+f_{ij}^{\text{r}}\right)\text{;}\;\left(f_{ji}=-f_{ij}\right)\text{;}\;f_{i}=\left(f_{i,x},f_{i,y},f_{i,z}\right)$$

using reduced time

(2) $$t=t^{\text{real}}/t_{\text{C}};\;t_{\text{C}}=\sqrt{m\cdot r_{\text{C}}^{2}/\left(k_{\text{B}}T\right)}$$

assuming equal mass *m* for all beads with position

(3) $$r_{i}=r_{i}^{\text{real}}/r_{\text{C}}$$

(in multiples of the cutoff radius *r*_C_) and reduced velocity

(4) $$v_{i}=v_{i}^{\text{real}}/\sqrt{k_{\text{B}}T/m}$$

under the influence of the (accordingly reduced) force

(5) $$f_{i}=f_{i}^{\text{real}}/\left(k_{\text{B}}T/r_{\text{C}}\right)$$

consisting of (short ranged) *conservative forces*, i.e., repulsion between non-bonded beads and a proper balance of repulsion and attraction between bonded segments

(6) $$f_{ij}^{\text{c}}=\{\begin{array}{cc} a_{ij}\cdot \left(1-r_{ij}\right)\cdot r_{ij}/r_{ij}-b_{ij}\cdot r_{ij} & r_{ij}<1\\ -b_{ij}\cdot r_{ij} & r_{ij}\geq 1 \end{array}\;b_{ij}=\{\begin{array}{cc} b & i\;\text{connected}\;\text{to}\;j\\ 0 & \text{otherwise} \end{array}$$

using the abbreviation

(7) $$r_{ij}=r_{i}-r_{j};\;r_{ij}=\left|r_{ij}\right|$$

*dissipative forces* (due to friction between particles)

(8) $$f_{ij}^{\text{d}}=\{\begin{array}{cc} -\gamma \cdot {\left(1-r_{ij}\right)}^{2}\cdot \left(\left(v_{i}-v_{j}\right)\cdot r_{ij}/r_{ij}\right)\cdot r_{ij}/r_{ij} & r_{ij}<1\\ 0 & r_{ij}\geq 1 \end{array}$$

and *random forces* (representing the thermostat together with the latter one)

(9) $$f_{ij}^{\text{r}}=\{\begin{array}{cc} \sqrt{2\gamma /\Delta t}\cdot \left(1-r_{ij}\right)\cdot \xi_{ij}\cdot r_{ij}/r_{ij} & r_{ij}<1\\ 0 & r_{ij}\geq 1 \end{array}$$

with *ξ*_*ij*_ being a Gaussian distributed random number and Δ*t* being the reduced time step. Since the force is dependent on particle velocities, a slightly modified Verlet velocity algorithm for time propagation is used. As stated above, parameters *a*_*ij*_ (giving the strength of repulsion in units of *k*_B_*T* with *k*_B_ being Boltzmann's constant and *T* representing absolute temperature) are dependent on the type of beads *i* and *j*, respectively, and read as *a*_PP_ if both beads belong to a polymer chain, *a*_PS_ = *a*_SP_ if one bead is a polymer segment and the other a solvent and *a*_SS_ for the interaction between two solvent beads. Simulation parameters have been chosen according to Ref. [33], i.e., Δ*t* = 0.04, *γ* = 4.5, total particle density *ρ* = 3, *a*_PP_ = *a*_SS_ = 25 and *b* = 4. For this contribution, all interaction parameters have been chosen as *a*_PS_ = *a*_PP_ to simulate athermal conditions and *a*_PS_ = 27.2 for theta solvents [36]. Results are based on 400,000–1,000,000 samples applying 5 time steps between two sampled systems corresponding to a total simulation time of 80–200·10^3^ *t*_C_. Statistical errors are obtained by the block averaging method [37] and are omitted in the diagrams if smaller than symbol size as well as in the case of data yielded from histograms.

## 3 Calculated properties

### 3.1 Shielding factor

As described in Ref. [8], the shielding factor *K*_*ij*_ is the rate constant *k*_*ij*_ of a bimolecular reaction with reactants *i* and *j* bound to polymer coils divided by the rate constant *k*_0_ of the same reaction without polymeric species involved. Given that the reaction occurs from the equilibrium pair configuration of two coils, *k*_*ij*_ and *k*_0_ can be directly identified as the probability of finding specific segments *i* and *j* of chains in contact without the need to simulate the reaction itself, i.e., the process of bond breaking and formation. Here, *i* and *j* have been chosen as ends of linear chains E, centers of star polymers C or the ends of star arms A, thus *K*_CE_, *K*_EE_, *K*_AE_, *K*_AA_ have been evaluated, see Scheme 1. The remaining possibilities *K*_CC_ and *K*_CA_ are excluded from analysis since they do not possess a counterpart in experimental polymerization schemes. In off-lattice simulations, a contact has to be defined as an approach of two point masses within a certain distance *r*_react_. A straightforward choice for *r*_react_ is the cutoff radius *r*_C_, because it roughly equals the mean length of one bond between two beads. Nevertheless, the impact of *r*_react_ on the shielding factor is also investigated in the current publication, see Appendix B. In the simulation, *K*_*ij*_ is calculated as given in equation (10) introduced in detail in Ref. [23]:

(10) $$K_{ij}=\frac{k_{ij}}{k_{0}}=\frac{\int_{0}^{r_{\text{react}}}\mathrm{d}r_{ij}4\text{π}r_{ij}^{2}g\left(r_{ij}\right)}{\int_{0}^{r_{\text{react}}}\mathrm{d}r4\text{π}r^{2}g_{0}\left(r\right)}=\frac{\sum_{0<r_{ij,k}<r_{\text{react}}}H\left(r_{ij,k}\right)}{\sum_{0<r_{k}<r_{\text{react}}}H_{0}\left(r_{k}\right)}$$

**Fig. 1:**
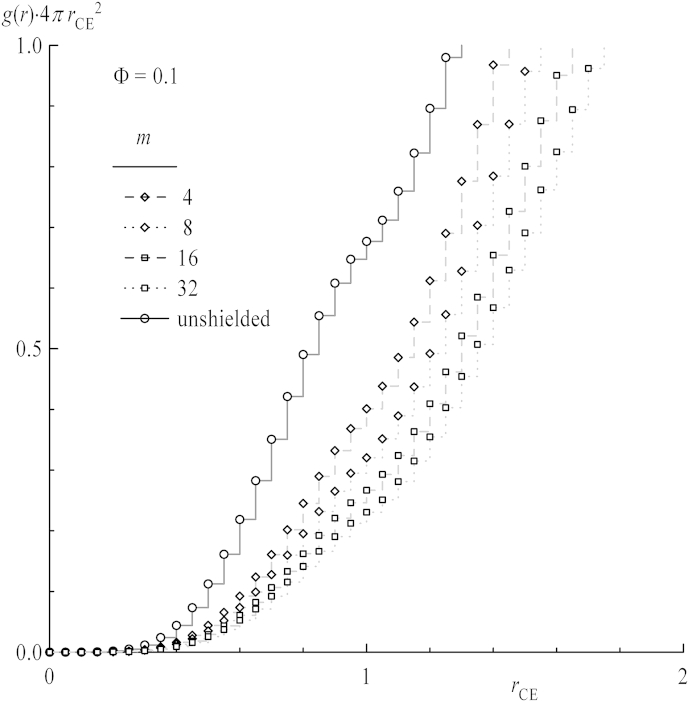
Examples of DPD frequency plots for distances between the center of stars and the ends of linear chains for selected chain lengths *m* at a concentration of *Φ* = 0.1. The full line depicts the unshielded monomer–monomer (reference) situation.

Here, *H*(*r*_*ij*,*k*_) is the frequency of distances between reactive centers *i* and *j* located in different coils in a spherical shell within radii *r*_*k*_ and *r*_*k*+1_ = *r*_*k*_ + Δ*r*. *H*_0_(*r*_*k*_) is the frequency of distances between single beads calculated in a simple fluid of free DPD particles. The terms *g*(*r*_*ij*_) and *g*_0_(*r*_*k*_) are the respective pair distribution functions. Fig. 1 shows the plots of *H*(*r*_CE,*k*_) for different chain lengths *m* for the overall concentration *Φ* = 0.1 and the reference frequency plot *H*_0_(*r*_*k*_). The shielding factor *K*_*ij*_ is expected to take values from 0 for perfect shielding to 1 for unshielded (free) reactants as the respective values *k*_*ij*_ of polymers are below *k*_0_ of free beads.

### 3.2 Size of DPD stars and chains

The average size of simulated polymer coils is evaluated by calculating the square radius of gyration *s*^2^ which is the mean square distance of all particles of a single star (or chain) from its center of mass. The mean over the whole ensemble and time is then ${\langle s^{2}\rangle }_{\text{str}}$ (or ${\langle s^{2}\rangle }_{\text{lin}}$), the mean square radius of gyration of stars (or linear chains). Analogously, $\langle r^{2}\rangle$ denotes the mean square distance between the center of a star and the terminal beads of its arms, and the end–end distance $\langle h^{2}\rangle$ is the mean square distance between terminal beads within chains or stars.

## 4 Results

### 4.1 Characterization of the systems

#### 4.1.1 Scaling behavior

In our calculations values of the edge lengths of cubic simulation boxes have been chosen as $L\approx 9\cdot {\langle s^{2}\rangle }_{\text{str}}^{0.5}$ which roughly resembles 4.5 times the mean radius of a pair formed by two interacting stars in order to avoid the interaction of periodic images [38]. Thus, depending on the average size of pairs of the solutes, the edge lengths of the simulation boxes were applied in the range from 14 *r*_C_ to 53 *r*_C_. The values for the radii of gyration have been taken from former works [36,39], addressing systems of stars and chains in the limit of infinite dilution. The edge lengths were held constant for each system with a specified chain length for simulations over the whole concentration range, since coils are most expanded at vanishing concentration *Φ* [36], thus ensuring that boxes are always large enough for any *Φ* > 0 chosen. One simulation system consists of star-branched chains having *F* = 4 arms, with *m* = 4, 6, 8, 12, 16, 24, 32, 48 beads per arm and linear chains with the number of beads *m* as one arm. This corresponds to the idea of one free detachable arm as depicted in Scheme 1. Therefore, the total number of segments for one star is *P*_str_ = 4*m* + 1, having one additional bead interconnecting its four arms, and for one linear chain simply *P*_lin_ = *m*. The number of bonds per star and per chain accordingly read *p*_str_ = *P*_str_ − 1 = 4*m* and *p*_lin_ = *P*_lin_ − 1 = *m* − 1. With the number of stars *N*_str_ and chains *N*_lin_ in a box the total mass concentration can be calculated as the ratio of the number of polymer beads and total beads (polymer + solvent). In this way *Φ* corresponds to a number density

(11) $$\Phi =N_{\text{pol}}/N_{\text{total}}=\left[N_{\text{str}}P_{\text{str}}+N_{\text{lin}}P_{\text{lin}}\right]/\rho L^{3}$$

taking values *Φ* ∈ [0, 1]. The density *ρ* is set to $3/r_{\text{C}}^{3}$ in this contribution. The influence of the ratio of chains and stars on the results is rather small (see Appendix A). Thus, in most cases *N*_str_ = *N*_lin_ has been chosen for convenience as well as to optimize the number of contact possibilities in order to improve statistical significance. The concentration range has been covered by performing calculations at *Φ* = 0.025, 0.05, 0.1, 0.2, 0.3, 0.4, 0.5, 0.6, 0.7, 0.8, 0.9, 1.0. This has been achieved by setting these values as target concentrations and then, before starting a simulation, filling the box with the closest possible number of polymers yielding the respective concentration since *N*_str_ and *N*_lin_ are restricted to integer numbers. After placement of the coils as self avoiding random walk chains at random positions, the remaining solvent beads were randomly distributed in the boxes, avoiding extremely close inter-particle distances (<0.2  *r*_C_).

In order to investigate whether the global properties of stars and chains are affected by the heterogeneity of the system (as there are always stars and chains in one box) scaling and its concentration behavior have been evaluated. This also yields useful information whether box sizes and chain lengths have been chosen large enough.

**Fig. 2:**
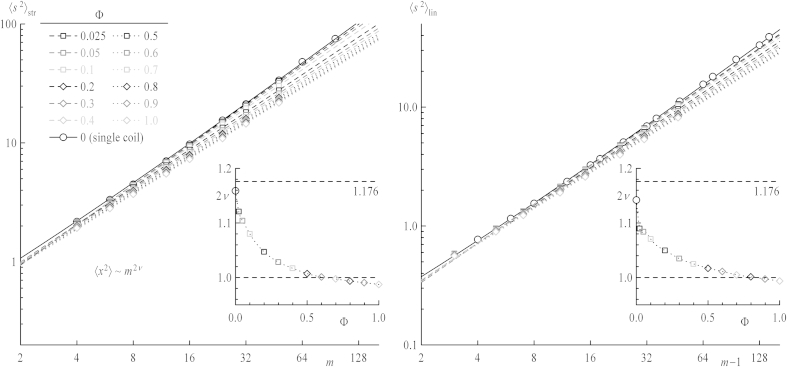
Log–log plots of mean square radii of gyration of stars (left) and chains (right) versus number of bonds per arm for all concentrations *Φ*. Dashed lines are linear fits and the full line includes corrections to scaling from renormalization group theory for short chains at high dilution. Insets show the obtained slopes (i.e., exponents) as a function of concentration. Horizontal dashed lines indicate theoretical values for infinite dilution and melt, respectively.

In Fig. 2 the log–log plots of $\langle s^{2}\rangle$ versus *m* for stars (left) and versus *m* − 1 for linear chains (right) are depicted to ensure that the systems – concerning both chain length and box size – are chosen large enough to yield reasonable scaling according to $\langle s^{2}\rangle =C\cdot p^{2\nu }$ which is valid in the limit of an infinite number of bonds *p* (*p* = *mF* ∼ *m* for stars and *p* = *m* − 1 for linear chains). Slopes of linear fits – dashed and dotted lines – in this representation reveal the exponents of $\langle s^{2}\rangle =C\cdot p^{2\nu }$. For higher dilutions of the polymers the exponents converge to the value 1.176 independent of topology as predicted by renormalization group theory [40–42] and verified by experiments and simulations [43] for sufficiently long chains. In this theoretical framework corrections to scaling for shorter chains in the form of a series expansion of the scaling law can be applied for infinitely diluted chains. To our knowledge, there is no analogous correction for the whole concentration range. In bulk, 2*ν* is expected to take the value one because intramolecular excluded volume is completely compensated by intermolecular excluded volume, following the Flory prediction [26]. For intermediate concentrations the exponents smoothly vary between values for dilute systems and for the bulk, as seen in the insets of Fig. 2 for both stars and linear chains. These results are obtained by leaving out the two shortest chain lengths thus yielding $2v_{\text{str}}^{\text{bulk}}=0.987$ and $2v_{\text{str}}^{\text{bulk}}=0.994$. The data for single coils – with fits depicted as solid lines – are taken from our previous work [39].

#### 4.1.2 Concentration dependence of global properties

**Fig. 3:**
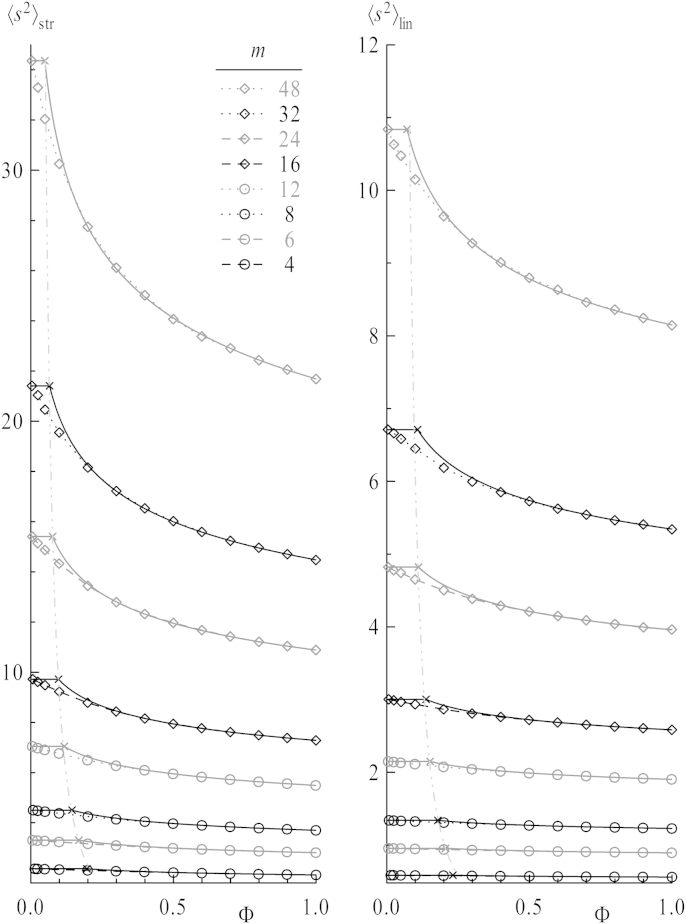
Mean square radii of gyration as a function of concentration for all chain lengths per arm *m*. Dotted and dashed lines are splines. Full and dashed-dotted lines are taken from the respective log–log plots of data (Fig. 4).

**Fig. 4:**
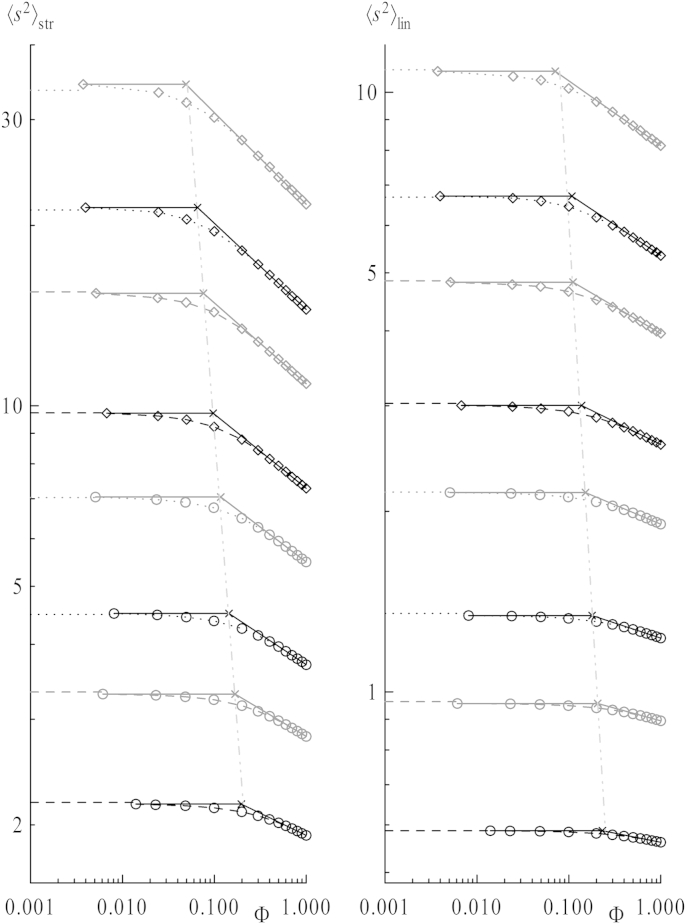
Log–log representation of the mean square radii of gyration $\langle s^{2}\rangle$ as a function of concentration for all chain lengths *m* from Fig. 3. Horizontal dashed and dotted lines indicate $\langle s^{2}\rangle$ at infinite dilution for stars and chains only. Inclined straight full lines result from linear fits of the highest four concentrations, horizontal full lines stem from data points of single pairs extended to the intersection points (marked with crosses) with the inclined ones. Dashed–dotted straight lines are yielded from linear fits of those intersection points.

In Fig. 3 mean square radii of gyration $\langle s^{2}\rangle$ of stars (left) and chains (right) are plotted as a function of the overall concentration *Φ* for 1:1 mixtures of stars and linear chains. For each chain length coils show the characteristic compression in size with increasing concentration, as the intermolecular excluded volume increasingly compensates the intramolecular one. This effect sets in as polymer coils start to interact when reaching a certain overlap concentration *Φ*∗. Theory predicts a scaling law for the concentration dependence of the radius of gyration in good solvent for regimes above *Φ*∗. It reads $\langle s^{2}\rangle ∼\Phi^{\lambda }$ with *λ* = −(1 − 2*ν*)/(1 − 3*ν*) ≈ −0.23 using the scaling exponent at infinite dilution *ν* = 0.588 [44,45]. Therefore, a log–log representation of the data is given in Fig. 4: The compression for higher concentrations is given by straight lines obtained by fits of the three highest concentrations *Φ* = 0.8, 0.9, 1.0 – depicted as full lines. In the normal plot (Fig. 3), these lines translate into curves. Dotted and dashed horizontal lines in Fig. 4 indicate the values for infinitely dilute systems, i.e., a single star or one linear chain in a simulation box. Surely, a certain concentration can be calculated for these systems by use of equation (11). But a single star or chain in a box has no interaction partner by design, so the concept of concentration is rendered useless in such a case. The only prerequisite is that the box is large enough to avoid self interactions. As the values of mean square dimensions remain constant under further enlargement of the box, it is straightforward to draw them at *Φ* = 0 in the normal plots or as horizontal lines in the log–log representations.

**Fig. 5:**
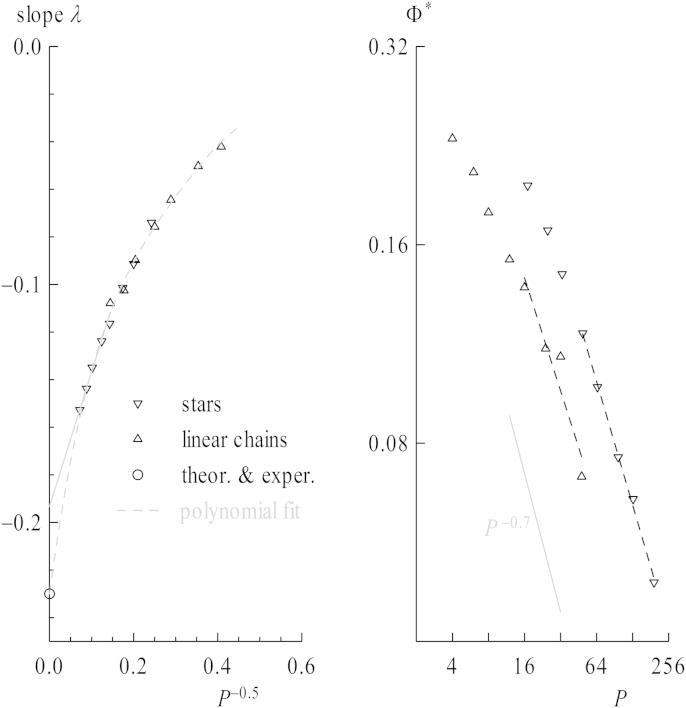
Left: slopes *λ* obtained from linear fits of coil compression (see Fig. 4) versus the inverse square root of number of beads for stars and chains. The straight line results from a linear fit of the 5 longest chains. Right: log–log plot of overlap concentrations (yielded from intersection points in Fig. 4) versus chain length for stars and chains. Straight lines are linear fits of the longest chains.

The slopes *λ* mentioned above are plotted in the left graph of Fig. 5 versus *P*^−0.5^, for an extrapolation toward an infinite number of bonds for stars and chains, respectively. Their values tend gradually toward the value −0.23 from theoretical predictions and experiments [46,47]. Taking into account that the scaling exponent of the chains does not perfectly reach the value 2*ν* = 1.176 but rather values 2*ν* ≈ 1.15, as seen in Fig. 2, *λ* ≈ −0.20 in our case. Note that the polynomial fit is forced to pass the point {0, −0.23} and acts as a guide for the eye only. The straight line is the result of a linear fit of the highest 4 chain lengths in this representation.

Mostly the overlap concentration of polymer chains *Φ*∗ is defined as the concentration at which a given space is filled densely by blobs spanned by non-interacting coils. It is reached when the overall concentration equals the density within one coil. Therefore $\Phi^{*}=P/V_{\text{coil}}=P/\left(4/3\text{π}{\langle s^{2}\rangle }^{3/2}\right)$ when using the radius of gyration as a measure for a spherical coil's size, and *P* being *P*_str_ or *P*_lin_. Similar definitions make use of $\langle h^{2}\rangle$ or introduce a factor $\text{π}/\left(3\sqrt{2}\right)\approx 0.74$ to take into account the volume occupation for close packing of spheres. Nevertheless, the overlap concentration scales as *Φ*∗ ∼ *P*/*p*^3*ν*^ ≈ *p*^1−3*ν*^ ≈ *P*^1−3*ν*^ ≈ *P*^−0.7^ in the limit of infinite chain lengths with *p* ≈ *P*, which also holds true for number densities. In the right plot of Fig. 5 the chain length dependence of the overlap concentration is depicted, alongside the slope stemming from the theoretical exponent. Again the highest chain lengths scale similarly. Note that this crossover of global properties is not a sharp transition at *Φ*∗, at least not for the chain lengths evaluated. Also the overlap concentration was evaluated separately for stars and chains because the concentrations where properties change are of interest for each topology separately. Here, it has been determined in the log–log plot (Fig. 4) by the intersecting point of the horizontal line given by the values for infinite dilution and the inclined line calculated by the fit for *λ* at high concentrations. These points can already be fitted via a straight line in Fig. 4 since $\Phi^{*}∼P/{\langle s^{2}\rangle }^{3/2}$, see the dashed-dotted lines.

### 4.2 Shielding

**Fig. 6:**
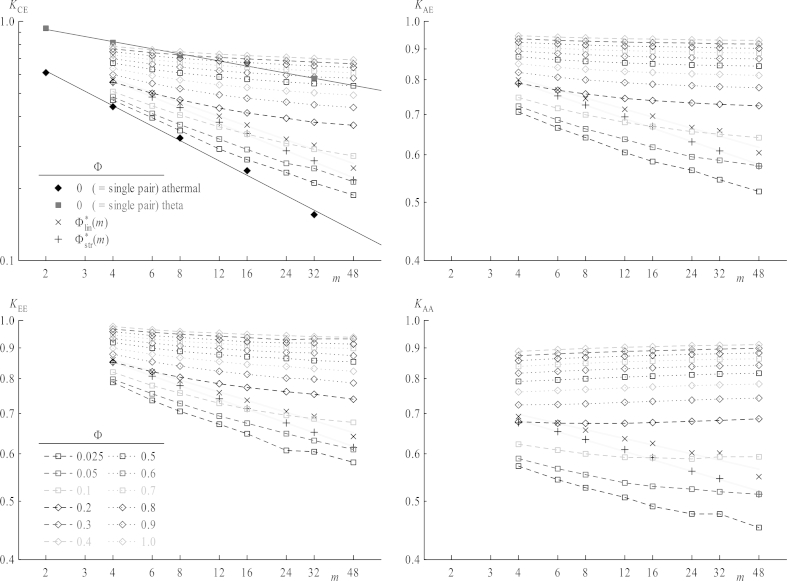
Shielding factors *K*_*ij*_ as a function of chain length *m* for all total concentrations and in a log–log representation. The straight lines in the top right graph connecting the diamond symbols are linear fits for infinitely diluted athermal and theta solutions respectively. Cross symbols mark shielding factors at the overlap concentration for each chain length, connected by straight lines as a guide for the eye.

The most important case is *K*_CE_, representing the central step in Z-RAFT polymerization, where also results of infinite systems, athermal and theta, are available. These results, taken from Ref. [23] are depicted as full symbols in Fig. 6. It has been shown in several previous works [8,20–23] that shielding factors in the limit *Φ* = 0 obey scaling laws

(12) $$K_{ij}=A\cdot m^{\varepsilon }\;\text{for}\;\Phi =0$$

yielding straight lines in log–log plots in Fig. 6. For short chains *K*_CE_ increases with concentration (i.e., shielding decreases) finally reaching approximately the same value in bulk as found for an infinitely diluted theta solution. For longer chains, however, the decrease of shielding with increasing concentration is much more pronounced leading to even larger *K*_CE_ values in bulk as compared to theta solutions, contrary to the assumption that the coil's behavior could coincide for these two regimes. Furthermore, data are not located on straight lines for *Φ* > 0 but exhibit some tendency toward a plateau value, at least for higher concentrations, corresponding to a chain length independent behavior in concentrated systems. Values for other shielding factors, *K*_EE_, K_AE_, K_AA_ also follow the trend to lower shielding factors at high dilutions, i.e., more pronounced shielding of the reacting sites for increasing chain length. The picture of a monotonic decay of *K*_*ij*_ for (nearly) isolated pairs of coils corroborates the idea that interpenetration is hindered more with increasing chain length. In addition the trend *K*_EE_ > *K*_AE_ > *K*_AA_ is observed, as ends of star chains are shielded by their other arms – see Ref. [22] for further discussion of this behavior. Also, values of *K*_CE_ are always below those of other *K*_*ij*_ which do not have a center directly involved as reactive site, because a property measured directly at the center is influenced by its additional excluded volume.

**Fig. 7:**
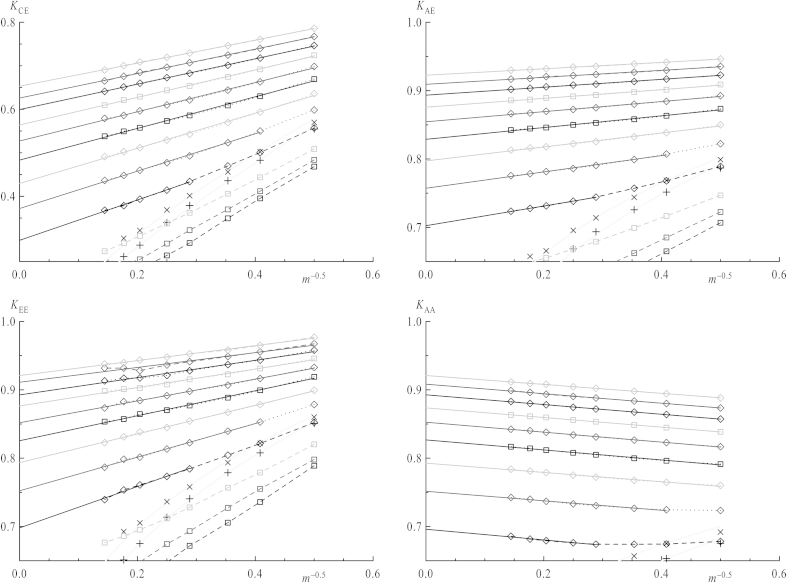
Shielding factors *K*_*ij*_ versus the inverse square root of the chain length *m* for all total concentrations. Symbols are as depicted in Fig. 6. Straight lines are extrapolations toward infinite chain lengths, using only data points above the overlap concentration for the respective chain length, which is given by cross symbols. The gray lines act as a guide for the eye.

**Fig. 8:**
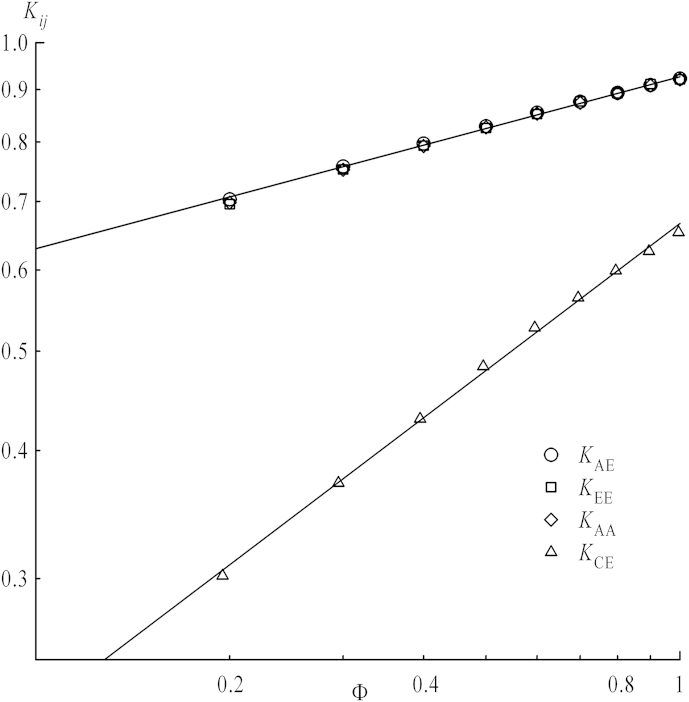
Log–log plots of shielding factors for infinite chain lengths, taken from the extrapolations in Fig. 7 for *m*^−0.5^ = 0.

**Fig. 9:**
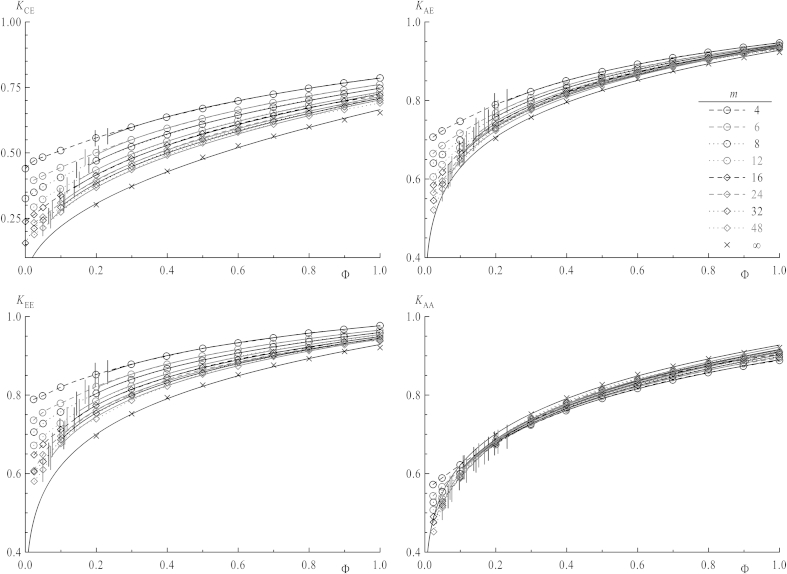
Shielding factors *K*_*ij*_ as a function of the overall concentration *Φ* for different chain lengths *m*. Vertical lines denote the overlap respective overlap concentrations. Full lines result from a fitted power law (see Fig. 10). Crosses mark the shielding factors extrapolated to infinite chain lengths.

In order to make results for different chain lengths better comparable the actual contact should be regarded with respect to the overlap concentration, which is a function of chain length. The overlap concentrations *Φ*∗ versus *m* has already been analyzed (see Fig. 5) which separate also shielding factors in those obtained below or above *Φ*∗, since properties related to interaction of coils are expected to change in the vicinity of *Φ*∗. The values *K*(*Φ*∗) in Fig. 6 are obtained from fitting functions in Fig. 9, which will be discussed later. When increasing the concentration above *Φ* = 0.025, the curves for all *K*_*ij*_ versus *m* do not obey the scaling law (with respect to chain length, equation (12)) anymore as it has been the case for dilute solutions. *K*_CE_, *K*_EE_, K_AE_ remain decreasing functions over the whole concentration range, but the chain length dependence of *K*_AA_, the only case concerning two stars, shows a different behavior for higher *Φ*: While below *Φ*∗ for their respective chain length *K*_AA_ tends to drop with increasing chain length for non-interacting chains, shielding factors above *Φ*∗ always increase. Thus, a minimum is observed near to *Φ*∗ in this latter case. As discussed above the presence of the center lowers the shielding factor for chain ends, and also *K*_AA_ values for very short arms are influenced by the center's excluded volume: Shielding factors increasing with *m* imply that the center's effect on the local density in the vicinity of ends vanishes for longer arms. Nevertheless, a diverging function for the shielding factor versus *m* is nonsensical as this would imply that reactions within coils of high chain lengths worked much faster than even between single monomers over a certain chain length. Thus, in all cases a plateau value should be reached for *m* ≫ 1 which may be evaluated by an extrapolation to infinite *m*, as seen in Fig. 7. All four types of *K*_*ij*_ versus *m*^−0.5^ are depicted, omitting data points below the respective values of the overlap concentrations. Indeed all extrapolated values of *K*_*ij*_ for *m* → ∞ concerning chain ends, regardless their connection to stars or linear chains, nicely coincide, whereas *K*_CE_, with a star center involved, is clearly shifted toward lower values. This indicates that at sufficiently large separations star centers do not interfere with end–end reactions. This is depicted in Fig. 8 in a log–log plot for *K*_*ij*_ of infinite chain length versus *Φ*∗. Their dependence on concentration can by fitted via a power law:

(13) $$K=B\cdot \Phi^{\mu }\;\text{for}\;\Phi >\Phi^{*}$$

**Fig. 10:**
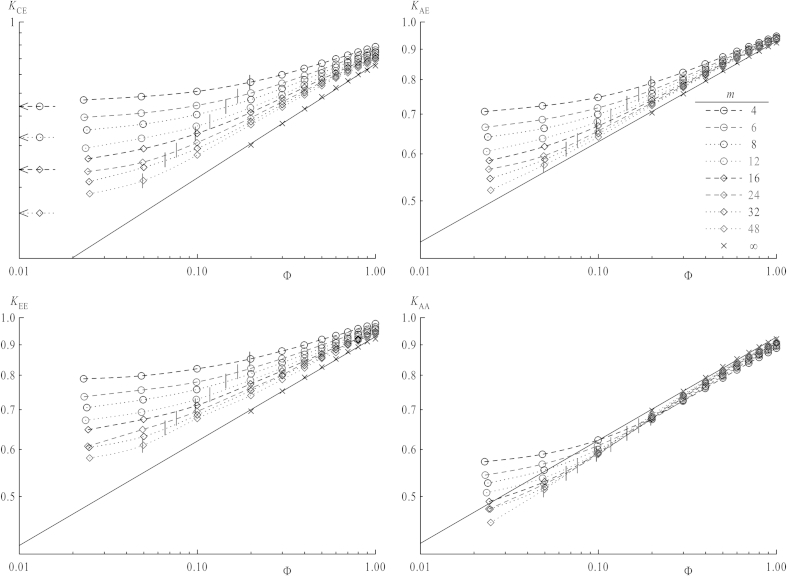
Same data as in Fig. 9 in a log–log representation. Vertical lines denote the overlap concentration *Φ*∗ of chains and straight inclined lines are fits of *K*_*ij*_ for concentrations above *Φ*∗. Crosses mark the shielding factors extrapolated to infinite chain lengths. Arrows on the top right graph denote data of the systems at infinite dilution (at *Φ* = 0, hence ln *Φ* = −∞).

Shielding factors as a function of concentration are shown in Fig. 9 in a conventional plot and in Fig. 10 in a log–log plot. In the latter one values of *Φ*∗ are depicted by straight horizontal lines for every chain length, which mark the beginning of concentration area to be fitted by equation (13). For all shielding factors between stars the higher overlap concentrations (i.e., those of linear chains) have been chosen since the systems always consist of stars and chains. As the transition is not a sharp one as already mentioned for mean square properties of coils, the first data point after *Φ*∗ also has been omitted from fitting. The dependence for infinitely long chains taken from Fig. 8 is also plotted in both the conventional and log–log plot (cross symbols) with the fit functions as full lines. By comparing plots of *K*_EE,_ *K*_AA_ and *K*_AE_, values for star ends are approaching the line for infinite chain length starting from lower values, while those with linear chain ends involved converge from higher values. Below the overlap concentration, shielding factors smoothly converge toward their respective value at infinite dilution, which is best shown in the log–log plots. For *K*_CE_ they are depicted by arrows on the right side. Since the overlap concentration itself converges to zero for infinite chain length, any properties at finite concentration should be described for these chains in a framework of interacting coils. As depicted in Figs. 9 and 10 the longer the chain, the broader is the concentration range where this power law with respect to *Φ* fits the curves. The power law describing shielding for an infinitely long chain nicely fits this picture because for *Φ*∗ = 0 equation (13) yields the value *K*_*ij*_ = 0, as it is for *m* → ∞ of equation (12) for the single pair.

## 5 Conclusions

In our previous simulations (MC + EE and DPD), shielding factors have been calculated between reaction sites located on several different positions of chains with various topologies, all in the limit of infinite dilution with the consistent result that the shielding factor as a function of chain length *m* is best described by power laws in every case. This work (DPD) concerns higher concentrations, for which a distinction has to be made between systems below and above the overlap concentration since the data obviously do not follow these power laws in the latter regime. As a first step analyses with regard to the mean square properties yield the required values of scaling exponents and the overlap concentration itself.

Shielding factors *K*_AE_, *K*_EE_ and *K*_CE_ versus *m* are decreasing functions for all *m* evaluated, regardless if below or above the overlap concentration, while values of *K*_AA_ which involve only terminal beads of star-shaped chain behave somewhat differently as they pass a minimum in the vicinity of the overlap concentration.

Nevertheless, for every concentration evaluated above the overlap concentration shielding factors for all contact schemes seem to converge to a constant value when increasing the chain lengths. Those cases not featuring a star center as reaction site yield the same extrapolation values, including those of *K*_AA_ which converge from lower values, starting from the aforementioned minimum. The concentration dependence of extrapolated values for reactions involving chain ends can then be described by one single power law, regardless the topology. Only for *K*_CE_ shielding is significantly more pronounced, fitted by a respective power law with different parameters.

## Figures and Tables

**Fig. A1:**
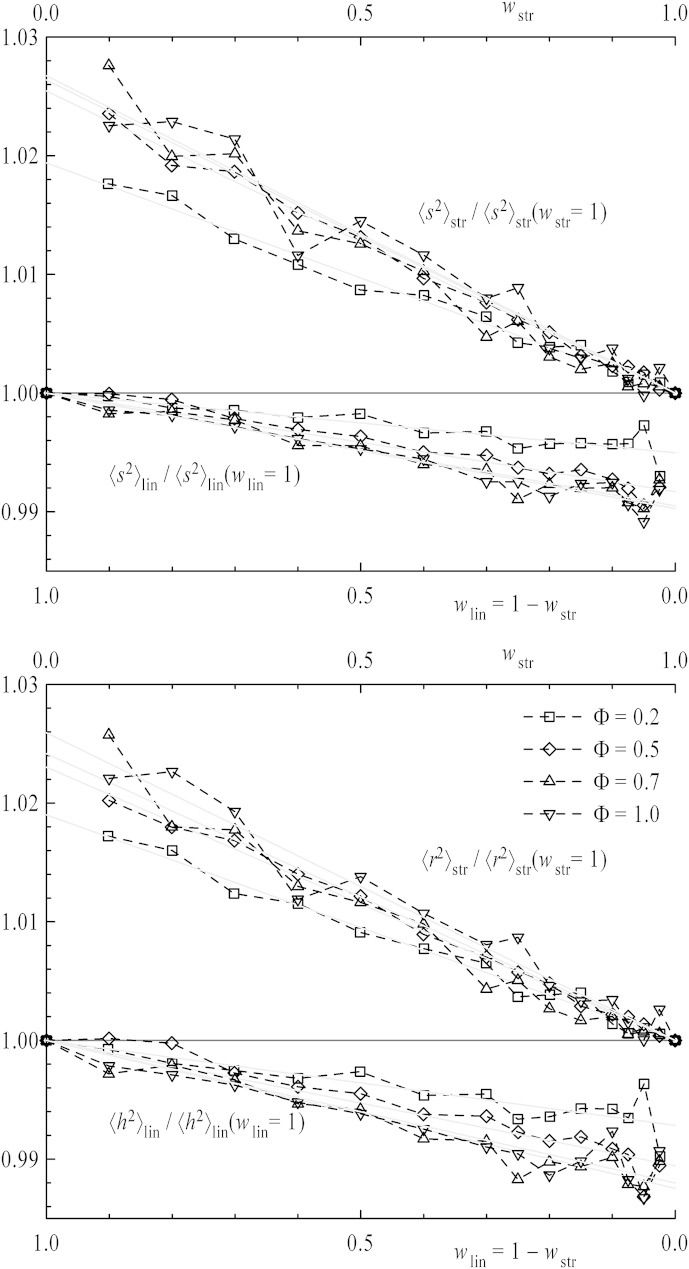
Top: mean square radii of gyration of stars and chains relative to their values of pure systems (star only, linear chains only) versus the mass fraction of stars (upper abscissa) which can be converted to the mass fraction of linear chains (lower abscissa). Data sets for stars and chains at each total concentration are fitted by straight lines. Bottom: the same plot and fit performed for mean square center end distances of stars and end–end distances of linear chains respectively.

**Fig. A2:**
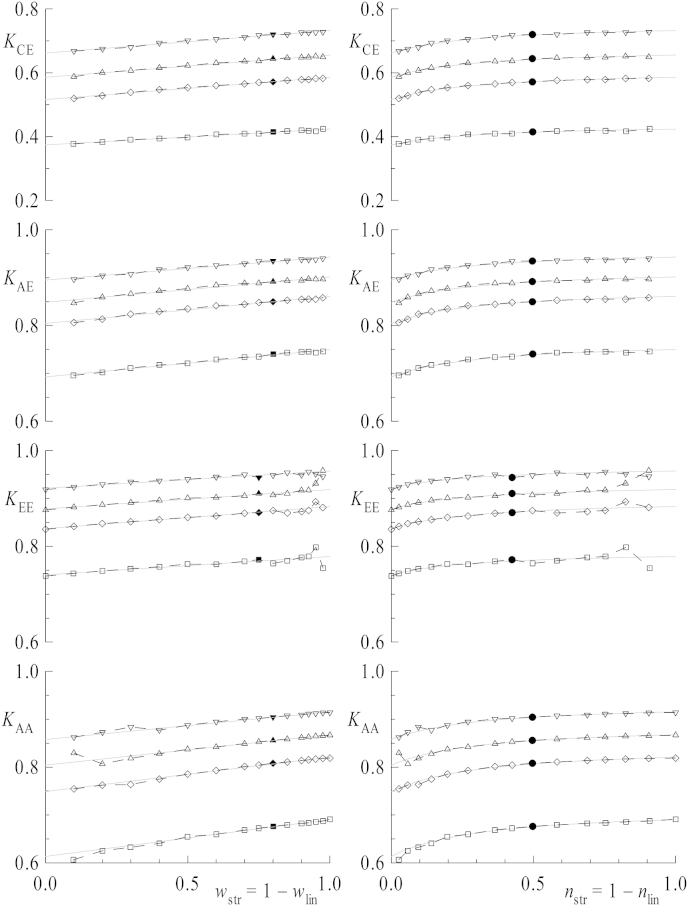
Left column: shielding factors *K*_*ij*_ versus mass fraction of stars for different total concentrations (as in Fig. A1). Straight full lines are linear fits. Right column: same data as a function of number concentration of stars and chains. Full lines are obtained by using the fit parameters from the left plot. Full symbols refer to *n*_lin_ = *n*_str_ = 0.5.

**Fig. A3:**
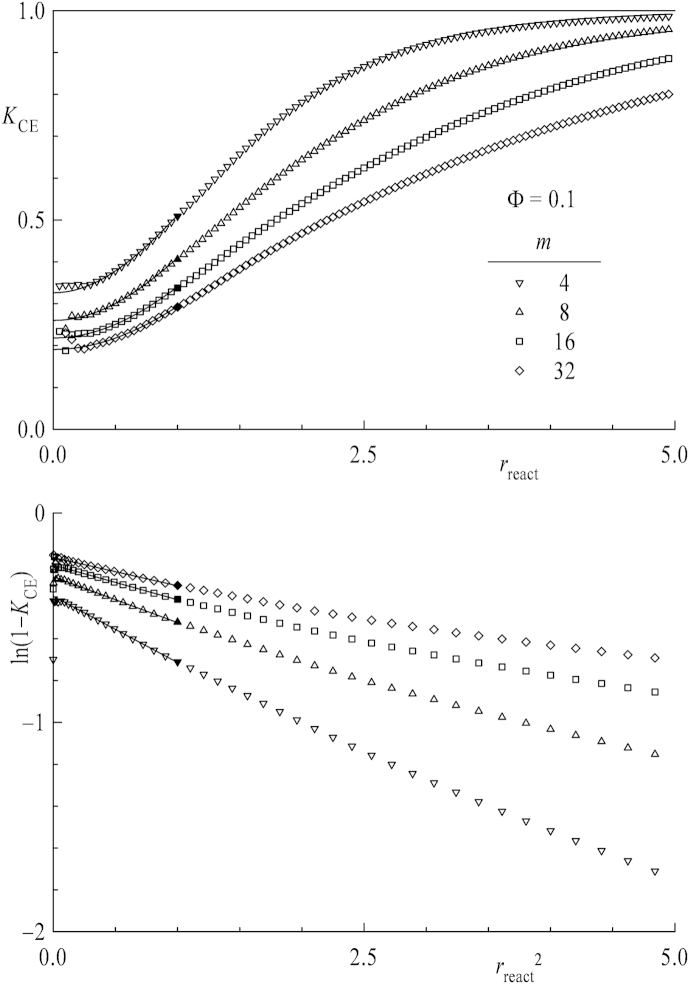
Top: shielding factors *K*_CE_ versus reaction radius *r*_react_ for some systems with chain length *m* and concentration *Φ* = 0.1. For radii <0.5 Gaussian fits are drawn as full curves. Bottom: plot of ln(1 − *K*) versus $r_{\text{react}}^{2}$ for a linearization of Gaussian curves. Straight lines are linear fits which yield the parameters for the Gaussian curves in the upper plot. Full symbols indicate *r*_react_ = *r*_C_.

## Appendix A Influence of the ratio of stars and chains:

The main reason for taking equal values for the number of stars and chains is merely for optimizing the number of possible contacts and, thus, increasing statistical significance as much as possible. Furthermore, it is in line with previous simulations in the limit of infinite dilution where the system under consideration clearly contains one star and one linear chain, i.e., a number ratio *n*_str_ = *n*_lin_ = 0.5. Nevertheless, different star/chain ratios could influence the properties of the systems including the calculated shielding factors. To estimate the amount of a possible effect the star/chain ratio has been varied for *m* = 16 and selected values of *Φ* and the results are based on the number as well as on the mass ratio.

(14) $$n_{\text{str}}=\frac{N_{\text{str}}}{N_{\text{str}}+N_{\text{lin}}}\text{;}\;n_{\text{lin}}=1-n_{\text{str}}=\frac{N_{\text{lin}}}{N_{\text{str}}+N_{\text{lin}}}$$

(15) $$w_{\text{str}}=\frac{P_{str}N_{\text{str}}}{P_{\text{lin}}N_{\text{lin}}+P_{\text{str}}N_{\text{str}}}\text{;}\;w_{\text{lin}}=1-w_{\text{str}}=\frac{P_{\text{lin}}N_{\text{lin}}}{P_{\text{lin}}N_{\text{lin}}+P_{\text{str}}N_{\text{str}}}$$

Fig. A1 shows changes of global properties for stars and chains normalized by their respective values from simulations of pure systems plotted against the mass ratio for different total concentrations. Mean square dimensions reveal a linear dependence on the mass ratio *w* of stars and linear chains. Obviously, both stars and chains are more compressed when shifting the ratio toward stars, as the presence of star cores deprives the system of free volume. However, these effects are very small amounting at most ca. 3%. As expected, $\langle r^{2}\rangle$ of stars as well as $\langle h^{2}\rangle$ for chains show the same relationship as $\langle s^{2}\rangle$.

In Ref. [48] effects of increasing concentrations of linear chains on one test star have been investigated experimentally and theoretically by applying a concentration dependent osmotic free energy contribution to Flory's equation for evaluating the scaling of a single polymer coil. By extrapolating ${\langle s^{2}\rangle }_{\text{str}}$ data in Fig. A1 to *w*_str_ = 0 we obtain this limiting case. Actually, as the dependence of size on the mixture ratio is quite small, the properties of stars and linear chains as a function of concentration behave nearly identically regardless of the mixture ratio. Thus, extrapolated values nearly coincide with data points in Figs. 3 and 4. Still, our findings coincide with Ref. [48]: At the overlap concentration of linear chains, a 10% drop of ${\langle s^{2}\rangle }_{\text{str}}$ is observed, and above *Φ*∗ values drop according to $\langle s^{2}\rangle ∼\Phi^{\lambda }$ as described before for mixtures with *w*_str_ = 0.5.

The respective shielding factors, *K*_*ij*_ are plotted in Fig. A2 as a function of mass ratio *w* (left) and number ratio *n* (right). The full symbols indicate the ratio used for the simulations as a function of chain length and the total concentration range. All data points are also following a linear relation versus *w* with increasing values for higher ratios of stars in all cases.

## Appendix B Influence of the contact range:

A last point to be discussed is the choice of the contact range. As already mentioned, above, the natural value within a segment model is of the order of the (average) length between two bonded beads, i.e., one lattice unit in lattice models or the cutoff radius in DPD. However, while for MC + EE positions are restricted to (integer) lattice positions leaving the neighbor positions as the only valid possibility, the soft potential used in DPD allows the realization of much smaller distances. Thus – although *r*_react_ = *r*_C_ should be the optimum value as discussed above – the influence of different reaction radii on shielding factors may be easily studied with DPD ranging from perfect overlap (*r*_react_ = 0) to also unrealistic large values corresponding to almost the whole polymer. By shifting the upper limit in the summation scheme (see equation (10)) different shielding factors *K*_*ij*_(*r*_react_) have been obtained as depicted in Fig. A3 (top) for *K*_CE_. It is a smooth function of *r*_react_ for all concentrations *Φ* and chain lengths *m*. Here systems with *m* = 4, 8, 16, 32 for *Φ* = 0.1 are shown as an example. Clearly, for reaction radii being large enough all curves approach *K*_*ij*_ = 1 because the corresponding pair distribution functions take the value one for large separations and the part contributing values <1 for smaller distances is then negligible in the summation. For the unshielded reference case *K*_*ij*_ is 1 trivially for all distances. Looking at smaller reaction radii the respective values converge toward a global minimum at *r* = 0. There the sphere spanned by the reaction radius degenerates to a point, rendering *K*_*ij*_ inaccessible numerically. Nevertheless data for small *r*_react_ can be used for an extrapolation: Assuming a Gaussian shape for close distances the curves have been linearized plotting ln(1 − *K*_CE_) versus (*r*_react_/*r*_C_)^2^, see Fig. A3 (bottom). Curves using parameters derived from linear regressions are also plotted in the normal representation. Values of *K*_*ij*_ decrease with decreasing *r*_react_, as the volume of overlaps increases. However, contrary to lattice models where *K*_*ij*_ = 0 throughout for *r*_react_ = 0 (as double occupancies are forbidden due to the excluded volume effect) *K*_*ij*_ values in DPD for *r*_react_ = 0 are far from zero due to the soft character of the potential and they obey the same scaling laws and fit functions introduced above. Thus, apart from the absolute values of *K*_*ij*_ similar behavior is obtained for a wide range of reaction radii. Nevertheless, further calculations should be exclusively performed using *r*_react_ = *r*_C_ not only for easy comparison with lattice data but in addition for physical reasons discussed above.
